# Older Latinx and African Americans’ Experiences of Civic Participation

**DOI:** 10.1093/geroni/igaa057.2582

**Published:** 2020-12-16

**Authors:** Laurent Reyes

**Affiliations:** Rutgers, The State University of New Jersey, Highland Park, New Jersey, United States

## Abstract

By 2030 Latinx and African Americans are expected to be the largest non-White groups of older adults. In the past 20 years, older adults’ civic participation has received considerable attention. However, until now most scholarship has focused on formal volunteerism and voting, activities that remain inaccessible to many marginalized groups. As a consequence, other civic activities are going unrecognized. The aim of this study is to understand how civic participation is experienced throughout the lives of 24 African American and Latinx adults 60+ living in New Jersey. Because civic participation is a concept that has many names and meanings depending on culture, language, and history I employ photo-elicitation techniques followed by in-depth interviews to understand civic participation through participants’ lens. Findings from this study can serve to improve conceptualizations and measurements of civic participation for future studies and inform efforts to strengthen civic participation among these populations. Part of a symposium sponsored by the Qualitative Research Interest Group.

